# Look Fear in the Eyes: The Influence of Emotion Regulation on Spider Size Estimation—A Pupillometry Study

**DOI:** 10.1111/psyp.70165

**Published:** 2025-10-27

**Authors:** Yahel Dror Ben‐Baruch, Noga Cohen

**Affiliations:** ^1^ Department of Special Education University of Haifa Haifa Israel; ^2^ The Edmond J. Safra Brain Research Center for the Study of Learning Disabilities University of Haifa Haifa Israel

**Keywords:** emotion regulation, perceptual bias, pupil, reappraisal, spider

## Abstract

The link between perceptual biases and fear has been extensively documented in recent years. For example, the size of spiders is overestimated among people with a high fear of them. While emotion regulation processes are known to reduce fear, it is yet unknown whether emotion regulation can also reduce fear‐related perceptual biases. This study examined the behavioral and physiological influence of cognitive reappraisal, an adaptive emotion regulation strategy, on spider size estimation among women with a high fear of spiders. Forty women with a high fear of spiders completed a trial‐by‐trial task with three conditions: (1) reappraising a negative image (reappraise‐negative), (2) passively viewing a negative image (watch‐negative), and (3) passively viewing a neutral image (watch‐neutral). Following each condition, participants estimated the size of either a spider or a butterfly depicted in a picture. Pupil size was tracked to assess arousal and regulation‐related processes. Results showed that reappraisal was associated with greater pupil dilation, reflecting heightened cognitive effort. Following reappraisal, participants reported the animals as smaller. Pupil size during the reappraisal assignment did not mediate the effect of condition on size ratings, suggesting that cognitive effort during the regulation did not predict the perceptual bias. Together, the findings suggest that instructed reappraisal can reduce perceptual biases associated with fearful stimuli, and that pupil dilation can serve as a physiological marker of emotion regulation. We discuss the implications of the findings for the understanding of the links between emotion regulation and perceptual biases.

## Impact Statement

This study shows that cognitive reappraisal can reduce perceptual biases in individuals with high fear of spiders. By combining behavioral measures of size perception with pupil diameter assessments, findings reveal that reappraisal leads to smaller spider size estimations and increased pupil dilation, indicating cognitive effort. These results highlight the role of emotion regulation in shaping perception and provide insight into arousal and regulation processes reflected in pupil responses.

## 1 Introduction

Perceptual biases linked to fear have been documented since the dawn of history. The Bible, for example, tells a story of the twelve spies who were sent to explore the land of Canaan before entering it. When they returned, they reported the land as flowing with milk and honey. They also described the inhabitants of the land as giants with unbeatable power, and added that they felt like grasshoppers beside them. As a result, the people were gripped by fear and panic, leading them to refuse to conquer Canaan (*Numbers 13*: 14–32). This example is supported by contemporary findings that show a connection between fear and perceptual biases (for review see MacIntyre et al. 2024). For instance, individuals with a fear of spiders tend to perceive spiders as larger compared to those without such fear (Leibovich et al. 2016). Despite substantial evidence linking fear to perceptual biases, the question of whether reducing fear using emotion regulation manipulation can mitigate these biases has yet to be explored. In this study, we aimed to address this question through a combination of a behavioral experiment and physiological measurements of pupil size. Specifically, we examined whether cognitive reappraisal, a well‐studied and effective emotion regulation strategy (Gross 2015; Ochsner et al. 2012), can reduce the size estimation of spiders among individuals with a fear of spiders. This was tested both behaviorally and using pupil dilation as a physiological marker of regulatory engagement and arousal.

Perceptual biases are cognitive distortions that lead individuals to perceive stimuli and objects subjectively, shaped by their emotions and beliefs (Dunning and Balcetis 2013). Several studies report a tight link between fear and perceptual bias. For example, people with a high fear of spiders perceived a spider's movement as faster when it moved toward them compared to when it moved away (Basanovic et al. 2018). Additionally, fear of spiders has been associated with attentional, expectancy, and perceptual biases toward spiders (Aue, Guex, et al. 2013; Aue, Hoeppli, et al. 2013; Basanovic et al. 2018; Ben‐Baruch et al. 2022; Riskind et al. 1995; Teachman and Woody 2003). These biases have been observed both in response to images of spiders (e.g., Leibovich et al. 2016; Ben‐Baruch et al. 2022) and during direct exposure to a live spider (Vasey et al. 2012). Similar effects were also observed for other fearful populations. For example, individuals who reported fear of heights while standing on a skateboard on a hilltop perceived the hill as steeper compared to individuals who stood on a box and did not report fear (Clerkin et al. 2009; Teachman et al. 2008). Similarly, individuals with social anxiety perceived the distance to other people as shorter compared to those without social anxiety (Givon‐Benjio and Okon‐Singer 2020). In light of this, the close link between perceptual biases and fear raises the question of whether fear underlies the perceptual bias. If so, regulating fear via emotion regulation strategies may reduce perceptual biases.

Emotion regulation refers to the set of processes involved in monitoring, evaluating, and modifying emotional reactions, particularly their intensity and duration, in order to achieve one's goals and well‐being (Aldao et al. 2010; Gross 1998). In this study, we focused on reappraisal, a cognitive emotion regulation strategy in which the person gives a different meaning to the situation in order to feel better about it (Gross and John 2003). People who use reappraisal more frequently experience more positive emotions and have better social interactions compared to people who use less adaptive emotion regulation strategies (Cutuli 2014; Gross and John 2003). In various lab experiments, it was found that reappraisal reduces the valence and the emotional arousal of negative stimuli (Buhle et al. 2014; Gross 1998; Gross and John 2003; McRae et al. 2012). This effect is associated with enhanced activity in brain regions associated with cognitive control and self‐monitoring, such as the ventrolateral prefrontal cortex (VLPFC), and reduced activity in areas related to emotional processing, such as the amygdala (Ochsner et al. 2002).

In regard to the current examination, reappraisal was found to reduce emotional responses to fear‐related stimuli (Langeslag and van Strien 2018). For example, a study that used electroencephalography (EEG), which measures the electrical activity in the brain, found that reappraisal reduces attention to snake and spider stimuli, as indicated by signal activity in the late positive potential (LPP) and early posterior negativity (EPN) components (Langeslag and van Strien 2018). In addition, Shurick et al. (2012) found that reappraisal of snake and spider images resulted in a decrease in experiential and autonomic fear experiences measured through electrodermal activity, which lasted 24 h after the reappraisal manipulation. Additionally, reappraisal moderated the subjective feeling of anxiety during a speech task among healthy individuals (Hofmann et al. 2009), as well as among individuals with math anxiety (Pizzie et al. 2020). Furthermore, studies show that a brief intervention of reappraisal among individuals with a high level of social anxiety reduced negative self‐beliefs and increased the frequency of reappraisal use (Kivity and Huppert 2016). Taken together, accumulating evidence suggests that reappraisal is effective in reducing the behavioral, neural, and physiological correlates of fear.

The current study examined whether reappraisal can reduce a perceptual bias associated with fear. Specifically, we tested whether an instructed reappraisal assignment can reduce the size estimation of spiders among individuals with a high fear of them. For that purpose, we used both behavioral assessment of size ratings, as well as a physiological measure of pupil dilation. Pupil dilation serves as an indicator of sympathetic arousal (Samuels and Szabadi 2008). Specifically, when encountering negative stimuli, the sympathetic system is activated, and this activation is reflected in the dilation of the pupils (Bradley et al. 2008; Kret et al. 2013; Samuels and Szabadi 2008). This dilation is an indication of activity in the locus coeruleus (LC), a group of neurons in the central nervous system that controls arousal and autonomic functions.

In recent years, numerous studies have used pupillary response as a measurement of emotional processes and regulation (e.g., Bradley et al. 2008; de Voogd et al. 2016; Hepsomali et al. 2017; Leuchs et al. 2017; Morriss et al. 2015; Nagai et al. 2002; Visser et al. 2013, 2016; Wiemer et al. 2014, 2021). Specifically, various studies have found that the pupil is more dilated in response to negative vs. neutral stimuli (e.g., Bradley et al. 2008; Kret et al. 2013). These effects have been observed using various stimuli, such as facial expressions (Hepsomali et al. 2017), pictures of various contents (e.g., Lang et al. 2008), as well as in response to movies (Raiturkar et al. 2016; Tarnowski et al. 2020) and pictures of spiders (Hoehl et al. 2017).

In addition to pupil dilation following emotional arousal, the pupil also dilates during emotion regulation (Kinner et al. 2017; Maier and Grueschow 2020, 2021; Ortigosa et al. 2023; Scheffel et al. 2021; Wiemer et al. 2021). This pupil dilation is probably due to recruitment of cognitive control processes, what researchers termed *regulatory engagement* (Kahneman and Beatty 1966; van der Wel and van Steenbergen 2018). For example, Maier and Grueschow (2021) found that increased pupil size indicates successful emotion regulation and can predict performance in self‐control tasks, such as choosing healthier foods (Maier and Grueschow 2020). Similarly, Ortigosa et al. (2023) observed greater pupil dilation in the reappraisal of cockroach images compared to a no‐regulation condition. Pupil dilation has also been observed with other emotion regulation strategies besides reappraisal (e.g., Kinner et al. 2017; Nasso et al. 2022).

The current study examined whether emotion regulation using cognitive reappraisal can reduce perceptual biases. Specifically, we investigated whether the size bias for spider pictures observed in people who are afraid of spiders can be modulated by the use of cognitive reappraisal. Despite accumulating evidence showing a link between perceptual biases and fear and anxiety (for review see MacIntyre et al. 2024), together with studies that show a reduction in fear following emotion regulation (e.g., Buhle et al. 2014; Diekhof et al. 2011; Hermann et al. 2013; Hofmann et al. 2009; Langeslag and van Strien 2018; Moustafa et al. 2017; Olatunji et al. 2017; Wiemer et al. 2021), to the best of our knowledge, the question of whether instructed emotion regulation can alter size bias was not examined thus far. Therefore, we measured whether cognitive reappraisal influenced the size bias previously found for spider stimuli among women with a high fear of spiders. Participants with a high fear of spiders conducted a trial‐by‐trial experiment that included two assignments in each trial: (1) observing a picture with/without regulation, and (2) reporting the size of an animal (spider/butterfly) on a visual analog scale. Therefore, the task included three conditions of regulation: a negative picture and a reappraisal cue (reappraise‐negative), a negative picture and a watch cue (watch‐negative), and a neutral picture and a watch cue (watch‐neutral). Following each regulation instruction, participants were asked to rate the size of a spider, for which they typically have a size bias, or a butterfly, which is considered a neutral stimulus. Importantly, based on previous task design (Ben‐Baruch et al. 2022; Leibovich et al. 2016), participants were instructed to estimate the real‐life size of the animal using a scale ranging from 1 to 100, representing the conceptual size of the animals rather than their actual size on the screen. To facilitate accurate judgments, the scale was anchored with a ladybug on the left end and a pigeon on the right end, serving as visual size references. Alongside the behavioral experiment, pupil diameter was measured throughout the experiment to assess sympathetic arousal.

Regarding the behavioral results, we expected to replicate prior findings showing that participants with a high fear of spiders rate the spiders as larger than the butterflies (Leibovich et al. 2016; Ben‐Baruch et al. 2022). Furthermore, we predicted that following the reappraise‐negative condition, participants will show lower size estimation of spiders as compared to the watch‐negative condition. Regarding pupil size, we expected to replicate prior findings that show an increase in pupil dilation following the reappraise‐negative vs. the watch‐negative condition (Kinner et al. 2017; Langer et al. 2020; Maier and Grueschow 2021; Martins et al. 2018), probably due to recruitment of cognitive resources associated with regulation engagement (Maier and Grueschow 2021; Peinkhofer et al. 2019). Furthermore, based on prior findings (Leuchs et al. 2017), we anticipated an effect of animal type on pupil dilation, with greater pupil dilation following a spider stimulus compared to a butterfly stimulus due to heightened arousal. Additionally, an exploratory analysis was made to test whether pupil size during the regulation phase (representing regulatory engagement) would mediate the relationship between task condition (watch‐negative/reappraise‐negative) and spiders' size ratings.

## 2 Materials and Methods

### 2.1 Participants

The study was approved by the institutional review board of the Faculty of Education, University of Haifa (No. 059/19). All methods were carried out in accordance with standard human research ethics guidelines and regulations (Declaration of Helsinki). Written informed consent was obtained from the participants. Since more women report fear of spiders than men (Okon‐Singer et al. 2011), and in order to avoid gender‐related effects, the study included only women participants. Based on Leibovich et al. (2016), participants included women who scored above 11 on the Spider Phobia Questionnaire (SPQ; Klorman et al. 1974), reflecting high fear of spiders. The SPQ is a 31‐item questionnaire that assesses fear of spiders (e.g., “I am careful when I buy fruit because bananas are known to attract spiders.”). The SPQ had a Cronbach's alpha of *α* = 0.89. The screening also included a few questions about fear of butterflies. Participants who reported a high fear of butterflies were not recruited.

### 2.2 Power Analysis

The sample size was based on a power analysis using G*Power (Faul et al. 2007) to assess a repeated measures ANOVA with two within‐subjects variables (i.e., condition × animal) with a power > 80% and a priori alpha set at *p* = 0.05. This analysis revealed a sample size of 28 participants. To account for dropouts, the sample consisted of 40 women (mean age = 24.625, SD = 3.670). All participants had normal vision and were asked to arrive at the experiment with no eyeliner or dark mascara. Data from four participants were removed from the physiological analysis as they had less than 30% valid trials in each condition (due to eye tracking issues). The behavioral experiment analysis included all participants.

### 2.3 Apparatus

Pupil size was measured using a video‐based desktop‐mounted eye tracker (EyeLink1000 Plus, SR Research, Ontario, Canada) with a sampling rate of 1000 Hz (1 ms intersampling time). The pupil area was determined using EyeLink's “centroid” algorithm. Stimulus presentation and data acquisition were controlled by Psychopy (Peirce et al. 2019) and were linked with the EyeLink Toolbox (Cornelissen et al. 2002), a software that supports the measurement of pupil dilation and eye movement. The experiment was presented on a 19‐in. ViewSonic monitor (Graphics Series G90fB), with physical screen dimensions of 52.5 cm (width) by 30 cm (height). Camera and head positions were fixed across participants, and participants were seated at a distance of approximately 58 cm from the screen. To maintain an accurate measure of pupil size during the task, participants were asked to keep their eyes fixed on the center of the screen and to avoid eye movements throughout the task. The neutral and negative pictures' size was 15 cm × 15 cm, resulting in a visual angle of approximately 14.8°. The size of the animal pictures was 20.5 cm × 15 cm, resulting in a visual angle of approximately 20.04° horizontally and 14.74° vertically.

### 2.4 Stimuli

#### 2.4.1 Negative and Neutral Pictures

Sixty‐four threat‐provoking pictures and thirty‐two neutral pictures were used in the experiment. These pictures were selected from the International Affective Picture System (IAPS; Lang et al. 2008) and the Nencki Affective Picture System (NAPS; Marchewka et al. 2014) database. The pictures were chosen based on their normative arousal levels (ranging from 1 = not arousing to 9 = highly arousing) and valence ratings (ranging from 1 = very unhappy to 9 = very happy). Additionally, five pictures were selected from the GAPED (Dan‐Glauser and Scherer 2011) database, whose arousal and valence ratings range from 0 to 100. To standardize the scale across all pictures, the GAPED ratings were divided by 10 to align with the 1–9 scale used for IAPS and NAPS pictures. Negative pictures were specifically selected for their normative high arousal and negative valence ratings (mean valence = 2.713; mean arousal = 6.545). Neutral pictures were selected based on their neutral valence and low arousal ratings (mean valence = 5.300; mean arousal = 4.149). Each picture was displayed only once during the experiment.

#### 2.4.2 Animals Pictures

The animal stimuli included 48 colored pictures of spiders from different species, and 48 colored pictures of butterflies from different species, taken from Pixabay 1.9, a website offering free pictures for commercial use. Spider pictures served as frightening stimuli, and butterfly pictures served as neutral stimuli. Each picture was displayed only once. All images were presented on the screen at a fixed and identical display size; however, the actual size of the animals (spiders, butterflies) within the images naturally varied across exemplars. This variability reflects real‐world differences among species and image composition and was not systematically biased toward any category.

#### 2.4.3 Brightness

To ensure that stimuli brightness did not affect our results, we used a custom MATLAB code to measure the average brightness of each picture type: neutral, negative, spider, and butterfly (see Table 1). An independent *t*‐test revealed no significant differences in brightness between the neutral and negative pictures, *t*(94) = −0.030, *p* = 0.976. Similarly, there were no significant differences in brightness between the spider and butterfly pictures, *t*(94) = 0.009, *p* = 0.993 (see Table 1).

**TABLE 1:** Mean brightness of the pictures in the experiment.

Type	*N*	M	SD
Neutral pictures	32	101.251	35.566
Negative pictures	64	101.452	28.930
Spider pictures	64	103.245	29.616
Butterfly pictures	32	103.191	28.794

*Note:* Mean luminance of the neutral and negative pictures and the spider and butterfly pictures. There were no significant differences between the picture types.

### 2.5 Design

The design contained two within‐subject factors: condition (reappraise‐negative, watch‐negative, watch‐neutral) and animal (spider, butterfly). Five additional trials included an emotion regulation cue and a scrambled picture. Except for the scrambled trials, the task included 96 trials in total (2 animals [spider, butterfly] × 3 conditions × 16 repetitions). Two sets of pictures with different valence were used; negative and neutral. Negative pictures were paired with either a reappraise or watch cue, while neutral pictures were paired with a watch cue only. The negative and neutral images were chosen randomly for each cue type, so each participant viewed a different set of images in each condition. Following each negative or neutral picture, a picture of a spider or a butterfly was displayed. The 5 scrambled pictures were presented alongside a reappraisal cue, to prevent effects related to anticipation (Maier and Grueschow 2021). Following these pictures, no spider or butterfly pictures were displayed. Trials were presented in a different random order for each participant.

### 2.6 Practice

Before beginning the experiment, participants completed a practice phase in order to guide them on how to perform the emotion regulation assignment, and the size estimation task. The practice phase included four guided trials, and nine trials of independent practice. In the guided trials, participants were asked to say their reappraisal out loud and the experimenter made sure that participants knew how to implement the strategy. The experimenter also made sure that participants understand that they should rate the perceived real‐life size of the animals and not their size on the screen. The practice included different animal pictures than those used in the actual experiment (snail, grasshopper, and mosquito).

### 2.7 Overall Procedure

**FIGURE 1:**
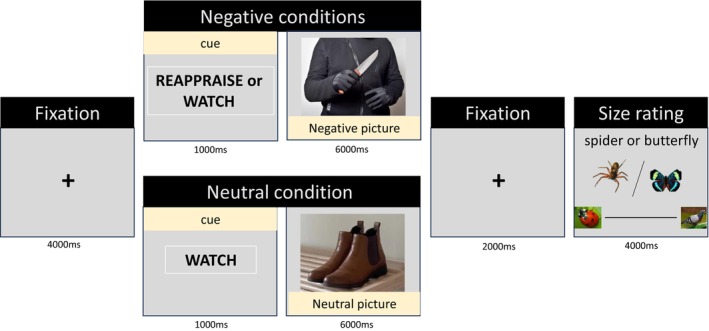
The task included 96 trials of three conditions: reappraise‐negative, watch‐negative, watch‐neutral. Each condition consisted of 32 trials, featuring 16 distinct pictures of spiders and 16 distinct pictures of butterflies. Following each condition, participants rated the size of a spider and a butterfly when a ladybug and a pigeon were used as a reference.

Individuals who were eligible to participate in the study based on the screening questionnaire (SPQ) were invited to the lab and performed the experiment in front of a computer screen. The experiment was a combination of the classic reappraisal task (e.g., Ochsner and Gross 2005) and a size estimation task (Leibovich et al. 2016; Ben‐Baruch et al. 2022). Therefore, the experiment included two parts in each trial: an emotion regulation assignment and a size estimation rating. The experiment started with a fixation cross for 4000 ms, and then a cue (reappraisal/watch) display for 1000 ms. Following the cue, a negative or a neutral picture appeared for 6000 ms. Participants were instructed to follow the instruction that appeared in the cue when watching the picture. The reappraisal cue was always followed by a threat‐provoking picture, while the watch cue was followed by either a threat‐provoking or a neutral picture. Therefore, the task included three regulation conditions: reappraisal‐negative, watch‐negative, or watch‐neutral. Following the picture, a fixation cross was presented for 2000 ms. After each regulation condition, participants were asked to rate the size of a spider or a butterfly in a picture display for 6000 ms. The size estimation rating was based on prior studies (Ben‐Baruch et al. 2022; Leibovich et al. 2016). Specifically, participants were instructed to estimate the animals' real‐world (conceptual) size rather than the size of the image on the screen. To assist in anchoring these judgments, a visual analog scale (VAS) was provided, ranging from a ladybug (left size) on one end to a pigeon on the other (right size), encouraging participants to consider real‐life dimensions rather than the physical size of the animal on the screen. Participants used the mouse cursor to make their ratings (see Figure 1). Before starting the actual task, participants completed a guided practice in which the experimenter made sure they understand and follow the task instructions. At the end of the experiment, participants were debriefed and thanked for their participation. They received monetary compensation worth $15 or course credit for their time.

#### 2.7.1 Reappraisal Instruction

The reappraisal instructions were based on those given in prior studies (Weinbach et al. 2022). Specifically, participants were instructed that “When the *reappraise* cue appears, we want you to change the way you think about the situation shown in the picture, or change the meaning of the picture for you, so that the situation in the picture will make you feel less stressful. For example: (1) You can think that the situation is not real (it's just a scene from a movie, they're pretending), (2) You can tell yourself that it looks bad but things will get better over time, (3) You can tell yourself that things are not as bad as they seem (it could be worse, at least it's not me in this situation). The goal is to change your thinking in order to feel better about the picture.”

#### 2.7.2 Watch Instruction

The watch instruction was based on prior studies (Doré et al. 2018; Weinbach et al. 2022). When the *watch* cue was shown, the participants were instructed to “allow yourself to feel the emotion that naturally arises in you in response to the pictures. Try not to change your feelings”.

### 2.8 Analytical Strategy

#### 2.8.1 Behavioral Analysis

Analyses of the main effects, interaction, and planned contrasts were conducted using JASP (JASP Team 2022). A repeated Analysis of Variance (ANOVA) was conducted with size estimation as a dependent variable and condition (reappraise‐negative, watch‐negative, watch‐neutral) and animal (spider, butterfly) as independent within‐subjects factors. *T*‐test *p*‐values are reported with Bonferroni correction for multiple comparisons.

#### 2.8.2 Pupil Data Preprocessing and Analysis

The preprocessing of pupil data followed a procedure consistent with methodologies employed in previous studies (Cohen et al. 2015; Hershman et al. 2024). Pupil data were preprocessed using CHAP (Hershman et al. 2017) to remove blinks and fill missing values using linear interpolation (Hershman et al. 2018). A baseline of 200 ms was used to define the time course, and pupil data was *Z*‐scored. This normalization was performed separately for each participant, using their entire time course (i.e., the mean and standard deviation were calculated across all trials for each participant). To analyze relevant time windows, we defined events of interest (negative/neutral picture, animal picture) and conditions (reappraise‐negative, watch‐negative, watch‐neutral/spider, butterfly) in the experiment. Trials with above 20% missing values were removed from the analysis. In order to examine the temporal differences between the conditions, we used CHAP (Hershman et al. 2017) to run a time‐series analysis. Specifically, a Bayesian paired‐sample *t*‐test was performed across the entire time window to examine the difference in pupil size between the conditions of interest. The Bayesian analysis provided Bayes factors (BF; Jeffreys 1961). A BF_10_ indicates evidence in favor of the alternative hypothesis relative to the null hypothesis (BF_01_). BF_10_ values above 3 are considered moderate evidence for the alternative hypothesis, values above 10 indicate strong support, values above 30 represent very strong support, and values above 100 reflect extreme support. This analysis was done for the picture time window (6 s of picture presentation following the regulation/watch cue), as well as for the animal time window (6 s of the spider or butterfly animal before size ratings were made).

Furthermore, a repeated Analysis of Variance (ANOVA) was conducted using JASP (JASP Team 2022) on the mean pupil size across the entire time course (picture or animal), with the significance level set at *α* = 0.05. Mean pupil size across the entire time course (picture or animal) served as the dependent variable, and condition (reappraise‐negative, watch‐negative, watch‐neutral, or spider, butterfly) served as the independent within‐subjects variable.

#### 2.8.3 Mediation Analysis

An exploratory mediation analysis was conducted to evaluate whether pupil size mediated the relationship between the experimental condition (reappraise‐negative vs. watch‐negative) and spider size estimation. Specifically, we conducted a causal multilevel mediation analysis using the R package “mediation” (Tingley et al. 2014). The analysis included only spider trials and data from 36 participants, yielding a total of 1477 valid observations (out of a maximum of 48 trials per participant). On average, 14.53% of pupil size data were missing due to blinks and eye‐tracking issues. The analysis incorporated participant as a random factor and examined whether mean pupil size (calculated during the picture presentation window) served as a mediator between condition and size ratings. We calculated the average causal mediation effect (ACME) and the average direct effect (ADE), using nonparametric bootstrapping with 5000 iterations. Complementary multilevel regression analyses were conducted using R to assess the links between pupil size and size ratings of spiders.

## 3 Results

### 3.1 Size Ratings

**FIGURE 2:**
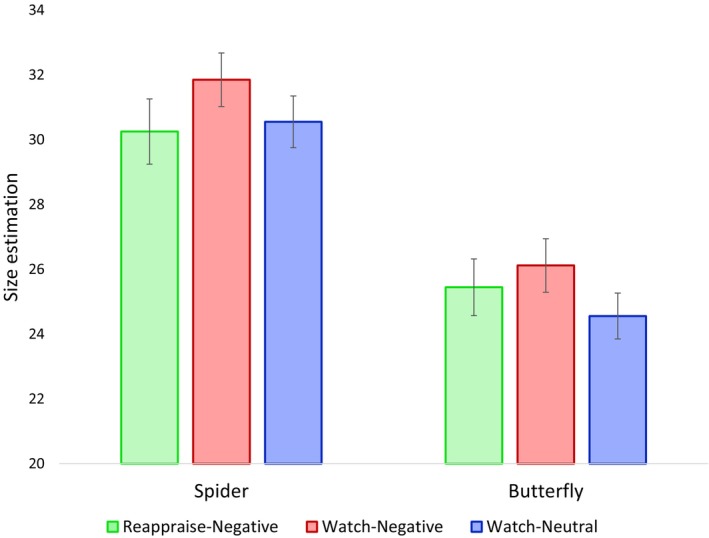
Spider and butterfly size ratings. Spiders were rated as larger than butterflies. Spiders and butterflies' size was estimated to be larger following the watch‐negative condition compared to the reappraise‐negative and the watch‐neutral conditions.

To examine the effect of condition and animal type on size ratings, we conducted a repeated measures analysis of variance (ANOVA) with two within‐subject factors: animal type (spiders, butterflies) and regulation condition (reappraise‐negative, watch‐negative, watch‐neutral). The results replicated previous findings (Leibovich et al. 2016; Ben‐Baruch et al. 2022), showing a significant main effect of animal type, with participants rating spiders as larger than butterflies, *F*(1, 39) = 11.203, *p* < 0.005, *partial ƞ* ^2^ = 0.223. Additionally, there was a significant main effect of condition, *F*(2, 78) = 3.344, *p* < 0.05, *partial ƞ* ^2^ = 0.079, indicating that size ratings differed across conditions. Specifically, participants rated both spiders and butterflies as larger in the watch‐negative condition compared to the other conditions, *t*(78) = 2.536, *p* < 0.005 (see Figure 2), suggesting that exposure to negative images increased perceived size for both animals. There was no difference in size ratings between the watch‐neutral and reappraise‐negative conditions, *t*(78) = 0.504, *p* = 0.615, suggesting that reappraisal was associated with similar size ratings to neutral trials. The interaction between animal type and condition was not significant, *F*(2, 78) = 0.804, *p* = 0.451, *partial ƞ* ^2^ = 0.020, indicating that the effect of condition on size ratings was similar for both spiders and butterflies (see Figure 2).

Specifically to our predictions, post hoc contrasts revealed that participants showed a tendency to estimate the animals as smaller in the reappraise‐negative condition compared to the watch‐negative condition, *t*(78) = 1.944, *p* = 0.055, and also smaller in the watch‐neutral condition compared to the watch‐negative condition, *t*(78) = 2.449, *p* < 0.05. No significant difference was found between the reappraise‐negative and watch‐neutral conditions, *t*(78) = −0.504, *p* = 0.615.

### 3.2 Picture Time‐Window

**FIGURE 3:**
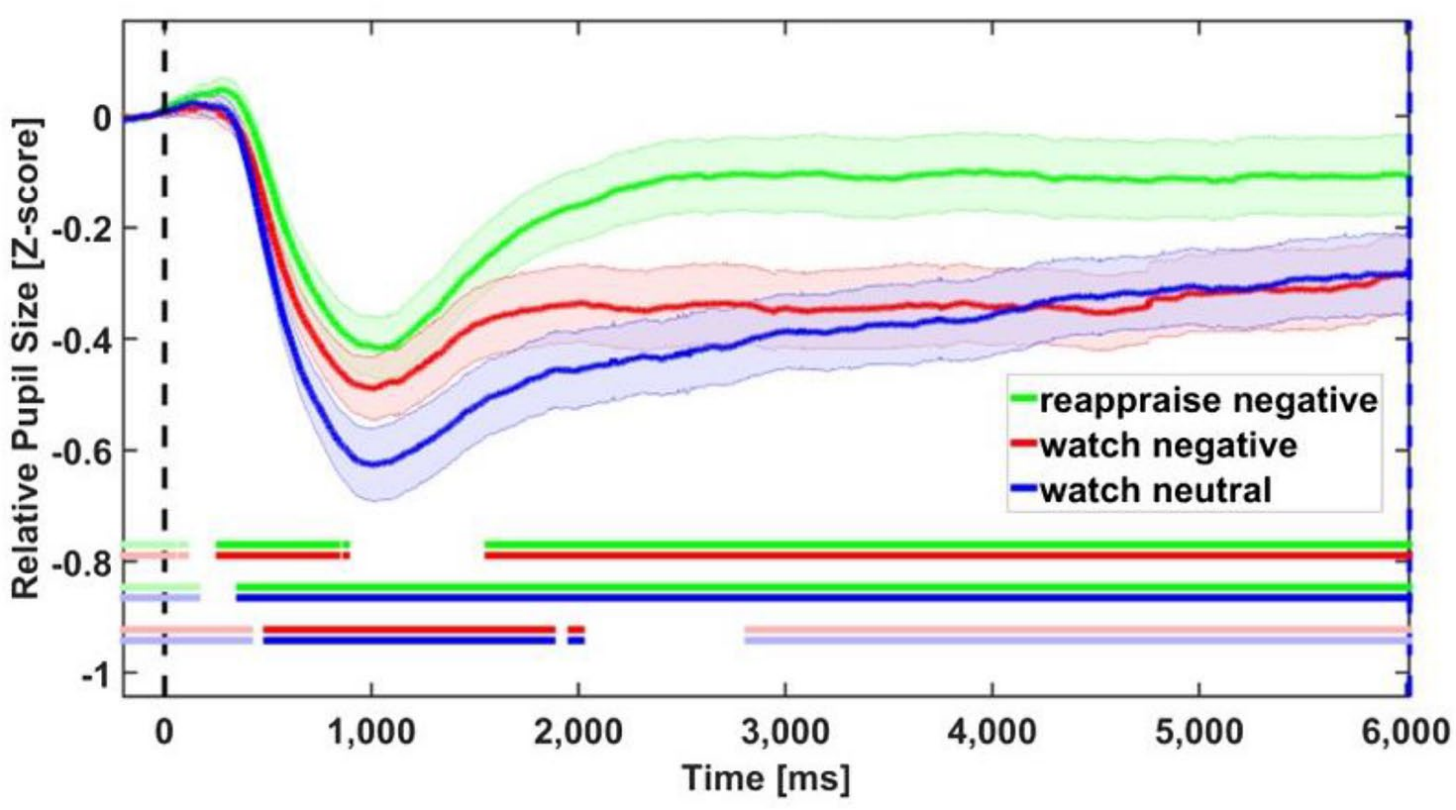
Results of pupil data in the picture time‐window. Mean relative pupil dilation (compared to the average of 200 ms prior to stimulus onset) is shown for all conditions: Reappraise‐negative (green line), watch‐negative (red line) and watch‐neutral (blue line). Each curve depicts pupil dilation changes over time, with shaded areas representing one standard error from the mean. Horizontal lines mark meaningful differences (BF_10_ ≥ 3) between conditions.

A repeated measures ANOVA with mean pupil size during the picture presentation revealed a main effect for condition, *F* (2, 70) = 16.209, *p* < 0.001, *partial ƞ* ^2^ = 0.317. Looking at the time course of pupil size during picture presentation (see Figure 3) revealed that the differences between the conditions appeared at about 1500 ms poststimulus onset and stayed until the end of the picture presentation (6000 ms). Specifically, in the reappraise‐negative condition, pupil size was larger than in the watch‐negative and the watch‐neutral conditions. This result is in line with prior findings of increased pupil dilation during a reappraisal assignment (e.g., Maier and Grueschow 2021, Yih et al. 2019; Ortigosa et al. 2023). The ANOVA findings were further supported by the Bayesian temporal analysis which showed a meaningful difference in pupil size between conditions BF_10_ ≥ 3 across most of the picture presentation time window (see Figure 3). Specifically, pupil size in the reappraise‐negative condition was greater than in the watch‐negative condition within the time window between 1500 ms and 6000 ms. Additionally, pupil size in the watch‐negative condition was greater than in the watch‐neutral condition within the time window between 500 ms and 2000 ms (see Figure 3).

### 3.3 Animal Time‐Window

**FIGURE 4:**
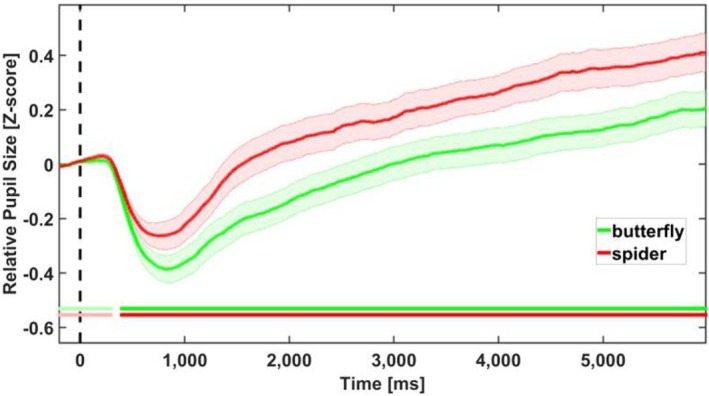
Results of pupil data in the animal time‐window. Bayesian analysis indicates a meaningful difference between animal pictures. Relative pupil dilation (compared to the average of 200 ms prior to stimulus onset) is shown for spiders (red line), and butterflies (green line). Each curve depicts pupil dilation changes over time, with shaded areas representing one standard error from the mean. Horizontal double lines mark meaningful differences (BF_10_ ≥ 3) between the spider picture and the butterfly pictures.

Mean pupil size during animal presentation was subjected to a paired‐sample *t*‐test with pupil size as the dependent variable. The results showed a main effect for animal, *t*(37) = −5.789, *p* < 0.001, indicating larger pupil size for spiders versus butterflies (see Figure 4). This finding was supported by the Bayesian temporal analysis, which showed a meaningful difference in pupil size between conditions (BF_10_ ≥ 3) in most of the animal time course (see Figure 4).

### 3.4 Causal Multilevel Mediation Results

The causal multilevel mediation analysis showed that pupil size did not mediate the relationship between condition (reappraise‐negative, watch‐negative, watch‐neutral) and spider size rating. ACME (indirect effect) = −0.075, 95% CI (−0.286, 0.121), *p* = 0.436, ADE (direct effect) = 1.318, 95% CI (−0.229, 3.016), *p* = 0.122, total effect = 1.243, 95% CI (−0.325, 2.949), *p* = 0.148, proportion mediated = −0.05, 95% CI (−0.987, 0.668), *p* = 0.508. Complementary multilevel regression analyses only on trials with spider pictures revealed that the effect of pupil size on size ratings was nonsignificant, *β* = 0.18, SE = 0.78, *t*(1436) = 0.23, *p* = 0.82, nor the interaction between pupil size and condition, *F*(2, 1436) = 0.14, *p* = 0.866.

## 4 Discussion

This study examined the effect of emotion regulation on perceptual biases, specifically whether cognitive reappraisal reduces the size bias toward spiders among individuals with a high fear of spiders. We conducted a trial‐by‐trial experiment with three regulation conditions: reappraise‐negative, watch‐negative, and watch‐neutral. After each regulation instruction, participants estimated the size of a spider or a butterfly. Physiological measurements were obtained by tracking pupil dilation, which allows capturing real‐time physiological activity reflecting emotional processes and regulatory mechanisms. Findings replicated prior studies showing that individuals with a high fear of spiders perceive spiders as larger than butterflies (Ben‐Baruch et al. 2022; Leibovich et al. 2016; Youssef et al. 2016). Importantly, as we predicted, reappraisal was associated with lower size estimation of spiders compared to the watch‐negative condition. This effect, however, appeared also for butterflies. Furthermore, we replicated prior results showing a larger increase in pupil size during the reappraise vs. watch conditions (e.g., Maier and Grueschow 2020, 2021), as well as during the viewing of spider vs. butterfly stimuli (Landová et al. 2023). An exploratory multilevel mediation analysis revealed that pupil size during the regulation phase did not mediate the link between regulation condition (reappraise, watch) and size ratings of the spiders.

The results of the study replicated prior findings showing that spiders are perceived as larger than butterflies (Ben‐Baruch et al. 2022; Leibovich et al. 2016). This effect aligns with previous studies demonstrating perceptual biases toward spiders among individuals who fear them (e.g., Li and Graham 2021; Youssef et al. 2016), as well as studies showing perceptual biases among other fearful populations, such as fear of heights (e.g., Teachman et al. 2008) and social anxiety (e.g., Givon‐Benjio and Okon‐Singer 2020). Interestingly, prior studies showed that even neutral stimuli that are perceived as threatening (such as a flying ball that might collide with a person) may be overestimated, likely due to a sense of threat or danger (Chen et al. 2016).

More importantly, and consistent with our prediction, the animals were rated as smaller in the reappraise‐negative vs. watch‐negative condition. This finding corresponds with findings showing that emotion regulation and other intervention methods may reduce distortion of thinking and perceptual biases (Li and Graham 2021; Tian et al. 2018; Yuan et al. 2020). For example, the size estimation of spiders among participants with a fear of spiders was smaller following the behavioral approach test (BAT) (Li and Graham 2021). In addition, an instructed reappraisal assignment effectively reduced distortions in time perception (Tian et al. 2018), as well as attentional biases toward spiders and snakes (Langeslag and van Strien 2018). Furthermore, distortions in self‐perception, manifested as negative self‐beliefs in individuals with social anxiety, were diminished following instructed reappraisal (Dixon et al. 2020). In fact, the reduction in size estimation of spiders following reappraisal suggests that reappraisal not only affects emotions, but may also influence emotion‐related effects. Indeed, similar findings were found for food preferences (Weinbach et al. 2022; Maier and Grueschow 2021), political attitudes (Halperin et al. 2013), and self‐related beliefs (Dixon et al. 2020). Our results are in line with these findings, demonstrating the power of emotion regulation, particularly reappraisal, in reducing cognitive distortions. Therefore, it can be assumed that the relationship between emotion and perception is a three‐stage process; it begins with an attitude or thought regarding the level of threat posed by the stimulus, continues with a feeling of fear, and leads to an overestimation of the stimulus. An example of this can be seen in Wilson et al. (2017), which demonstrates that stereotypes toward Black individuals cause a sense of threat, subsequently leading to an overestimation of their physical size. Given that the relationship follows a three‐stage process, it is possible to reduce perceptual biases by intervening at the first stage, such as by modifying specific perceptions or attitudes toward the threatening stimulus through psychoeducational intervention. For example, Kürümlüoğlugil and Tanrıverdi (2022) demonstrated changes in cognitive distortions among individuals with depression following psychoeducational treatment. Our study uniquely demonstrates that regulating fear unrelated to the threatening object can also mitigate cognitive bias. As mentioned before, this aligns with previous findings showing that reappraising threatening stimuli can influence unrelated cognitive processes, such as food preferences (Weinbach et al. 2022; Maier and Grueschow 2021) and political attitudes (Halperin et al. 2013). Based on this, we suggest that reducing fear itself can lead to a reduction in perceptual biases.

Interestingly, the size estimation of spiders and butterflies was higher in the watch‐negative condition compared to the watch‐neutral condition. Regarding spiders, these findings are consistent with previous research demonstrating the impact of emotional manipulations on the cognitive and perceptual performance of individuals with high anxiety (Jung et al. 2014; Keogh and French 1997). For example, mood manipulation through the presentation of negative stimuli to high‐anxiety participants led to a decrease in cognitive performance (accuracy and speed) compared to the control group (Keogh and French 1997). Furthermore, mood manipulation through feedback on task performance resulted in impaired performance among participants who received negative feedback (Jung et al. 2014), compared to neutral feedback. Additionally, the findings align with studies showing that threat manipulation affects visual perception and alters the size estimations of stimuli. For instance, simulating a ball moving toward a potential collision with a person led participants to perceive it as larger compared to a ball moving without the collision risk (Chen et al. 2016). In addition, presenting participants with threatening avatars and nonthreatening avatars led participants to perceive the threatening avatar as closer (Fini et al. 2020).

It makes sense that the manipulation of negative mood increased unpleasant feelings among participants and led to greater cognitive distortions, such as overestimation of the spiders' size. Regarding the butterflies, which are generally considered neutral stimuli, it is possible that the negative images presented prior to these stimuli induced heightened fear or emotional arousal, resulting in an overestimation of the butterflies' size compared to the neutral condition. Several studies suggest that neutral stimuli can be perceived as threatening when temporally associated with aversive cues (e.g., Amir et al. 2005; Mogg and Bradley 1998; Phelps et al. 2001). For example, increased amygdala activity, known to be involved in emotional processing, especially fear, has been observed in response to neutral stimuli paired with negative stimuli (Phelps et al. 2001). Additionally, individuals with high anxiety show attentional biases toward otherwise neutral stimuli (Mogg and Bradley 1998), and those with social anxiety tend to interpret ambiguous situations more negatively than individuals without social anxiety (Amir et al. 2005). Furthermore, heightened emotional arousal elicited by threatening images likely contributed to perceiving the butterflies as larger after viewing negative images, similar to findings that arousing films can alter time perception (Appelqvist‐Dalton et al. 2022). Taken together, presenting negative images, including threat‐provoking pictures and spider pictures, to women with a high fear of spiders may have increased fear and anxiety throughout the experiment, thereby heightening size perception not only for spiders but also for neutral stimuli such as butterflies.

In line with this, research on cognitive reappraisal's effects on ambiguous stimuli has shown that individuals who habitually use reappraisal in daily life tend to interpret emotionally ambiguous cues (e.g., surprised faces) more positively under stress compared to those who use reappraisal less frequently (Raio et al. 2021). Moreover, experimental induction of reappraisal was shown to increase positive interpretations of ambiguity (Neta et al. 2023). Based on these findings, it is possible to assume that the negative images preceding butterfly presentation rendered the butterflies emotionally ambiguous, leading participants to overestimate their size in the passive viewing–negative condition. However, reappraisal appears to reduce this negative interpretative bias, resulting in smaller size estimates for butterflies, similarly to its effect on spiders.

Our study used pupil dilation as a marker of both regulatory engagement and physiological arousal. Findings replicate prior results showing that reappraisal is associated with an increase in pupil dilation (Hamilton and Allard 2021; Maier and Grueschow 2020, 2021; Martins et al. 2018; Ortigosa et al. 2023; Scheffel et al. 2021), and align with our hypothesis, which posited that emotion regulation through reappraisal would recruit significant cognitive resources, leading to increased pupil diameter (Samuels and Szabadi 2008). Additionally, consistent with our prediction, analysis of pupil size revealed a larger pupil dilation when viewing spider images compared to butterfly images.

The increase in pupil dilation following the reappraisal condition aligns with findings from several studies that document similar effects. Pupil dilation during reappraisal has been consistently observed across a range of tasks, including those involving health‐related challenges (Maier and Grueschow 2020, 2021), fear ratings of cockroaches (Ortigosa et al. 2023), and assessments of perceived accountability (Yih et al. 2019). This response is linked to positive behavioral outcomes, such as enhanced self‐control (Maier and Grueschow 2021), reduced negative feelings toward cockroaches (Ortigosa et al. 2023), and decreased perceived responsibility (Yih et al. 2019). Similar effects have been noted in distinct populations. For example, clinically anxious adolescents demonstrated larger pupil dilation and a reduction in anxiety and negative emotions following reappraisal (De Witte et al. 2017). Older adults who implemented reappraisal rated memories more positively than younger adults. However, they exhibited greater pupil dilation, indicating that the process required more regulatory engagement (Hamilton and Allard 2021). Here, we used a time‐series analysis to understand the specific pattern of pupil dilation. It is important to distinguish between pupil dilation caused by emotional arousal and dilation resulting from the regulatory engagement of the reappraisal assignment. Our temporal analysis revealed that in the reappraisal condition, the pupil reached its maximum size within 1–3 s after the onset of the negative image. In contrast, under the watch‐negative condition, the pupil size remained stable within the first 1–2 s. The larger pupil dilation observed during the reappraisal condition may indicate the regulatory engagement involved in regulating emotions, and may reflect successful emotion regulation as found in prior studies (e.g., Maier and Grueschow 2021).

Regarding the increase in pupil dilation following spider pictures, there is substantial evidence that pupil dilation occurs in response to emotionally significant stimuli. This effect is evident for both images and sounds (e.g., Bradley et al. 2008; Cohen et al. 2015; Ferrari et al. 2016; Henderson et al. 2018; Kret et al. 2013; Partala and Surakka 2003; Vanderhasselt et al. 2014; Zekveld et al. 2018). Relevant to the current research, this effect was also observed on pictures of animals, such as crabs, scorpions, and snakes, and especially in response to spiders (Landová et al. 2023). In our study, the dilation of the pupil observed after watching pictures of spiders suggests significant emotional arousal, likely stemming from fear. Given that female participants who fear spiders were recruited, it is reasonable to infer that the spider images elicited fear, which was physiologically reflected in the pupils' dilation. The pupil dilation observed in response to spider images, combined with the study's behavioral findings indicating an overestimation of spiders, underscores the connection between fear and perceptual biases.

Contrary to our prediction, the multilevel causal mediation analysis did not yield a significant mediation effect, suggesting that pupil size did not mediate the relationship between regulation condition (reappraise, watch) and size ratings of the spiders. That is, the regulation engagement involved in the reappraisal task (as indexed by pupil dilation) did not predict the perceptual outcome. This result contrasts prior findings linking pupil dilation during reappraisal to behavioral outcomes, such as food preferences (Maier and Grueschow 2021). However, our study is the first to examine this link on a trial‐by‐trial level, offering a unique perspective on the immediate, within‐trial dynamics between regulatory engagement and perceptual judgment. Importantly, findings of the mediation analysis suggest that the execution of the reappraisal strategy itself, regardless of the effort invested, was sufficient to influence size ratings. Namely, that reappraisal can impact perceptual judgments even when regulatory effort is low, emphasizing the importance of strategy implementation itself over the magnitude of cognitive engagement. Alternatively, it is possible that the specific task design limited the ability to detect mediation effects. Trial‐level physiological measures, such as pupil size, are known to be relatively noisy (Nebe et al. 2023), and indeed, some trials were excluded due to missing pupil data. Increasing the number of trials or participants could have enhanced the statistical power to detect such effects. Importantly, the mediation analysis was exploratory in nature and was not the primary focus of the study. The study was powered to detect effects in the repeated‐measures ANOVA on size ratings, but not for the mediation analysis. Therefore, the null result in the mediation model should not be taken as definitive evidence for the absence of an indirect effect. Rather, it may reflect limitations in power. Future studies that employ more robust designs,for example by increasing the number of trials per participant or the overall sample,may be better suited to uncover the hypothesized mediation pathway.

A key strength of our study is the use of a trial‐by‐trial design in which both the emotion regulation task and the size estimation task occur within the same trial. This structure allowed us to directly examine whether the first task (emotion regulation) immediately influences the second (size perception), capturing the direct impact of reappraisal on perceptual judgments. Separating the two tasks into distinct blocks may obscure this immediate relationship, as emotional states may dissipate or become confounded with other factors between blocks. Importantly, the trial‐by‐trial design enhances sensitivity to moment‐to‐moment fluctuations in emotional and perceptual responses. Rather than averaging across trials, or relying on general condition‐level comparisons, our analysis focused on each individual trial, linking pupil dilation to the corresponding size rating. This allowed us to capture fine‐grained, intraindividual variability in regulation engagement and perceptual bias, a level of resolution that would be lost in block designs, which are more successful in capturing sustained effects. Overall, this design provides a more precise approach for investigating the transient effect of emotion regulation on perception. Although the trial‐by‐trial design has clear advantages, it may also involve the possibility of spillover effects between trials, that is, regulatory effort or emotional responses elicited in one trial may persist and influence responses in subsequent trials. Nevertheless, we took steps to minimize this risk, such as randomizing trial order and interleaving neutral and negative stimuli, as well as the addition of trials with scrambled images. Furthermore, fixation intervals lasted several seconds, allowing temporal spacing between trials. Lastly, as this is a within‐subjects design, any possible spillover would have occurred in all trial types and therefore cannot account for the observed findings.

Another strength of the current study lies in its use of negative images that were unrelated to the fear‐relevant stimuli. This design choice, which is standard in reappraisal research (e.g., Maier and Grueschow 2020; Weinbach et al. 2022), allowed us to elicit a general negative emotional response and assess regulatory processes in a context that is not confounded by direct emotional reactions to the target stimuli (e.g., spiders or butterflies). By having participants reappraise a separate unpleasant image before making size estimations, we were able to isolate the effects of emotion regulation on subsequent perceptual judgments. This feature underscores the unique contribution of our study. Specifically, it suggests that perceptual biases toward threatening stimuli can be modulated not only by reinterpreting the feared object itself, but also through broader emotion regulation efforts. Our findings imply that effective regulation does not necessarily require direct modification of the fear‐eliciting stimulus, but may occur via the establishment of a general regulated emotional state. This insight highlights the potential of nonspecific regulatory strategies to reduce fear‐related perceptual distortions, offering implications for both basic affective science and translational applications in anxiety treatment.

Some limitations of the present study should be acknowledged. The behavioral experiment involved estimating the sizes of spiders and butterflies under three conditions: reappraise‐negative, watch‐negative, and watch‐neutral. However, the study did not include emotional ratings following the reappraise or watch conditions. This design choice is consistent with previous studies examining the effects of cognitive reappraisal on cognitive distortions and decision‐making, such as in the context of food choices (e.g., Weinbach et al. 2022). These studies have consistently demonstrated the effectiveness of cognitive reappraisal in reducing negative emotional responses, as supported by participants' self‐reports (e.g., Dawel et al. 2023; Dixon et al. 2020; Goldin et al. 2019; Mohammed et al. 2021; Ochsner et al. 2012; Weinbach et al. 2022). While including subjective emotional ratings could have further confirmed task engagement, it would have significantly increased the duration and complexity of the experimental procedure. Our current design enabled participants to complete the task within approximately 50 min, making it feasible. Future research incorporating emotional and unpleasantness ratings could offer additional insights into how emotion regulation influences fear‐related perceptual biases.

Another limitation of the current design is the absence of a cognitively demanding control condition (e.g., a non‐emotional task such as mental arithmetic), which could help disentangle the specific effects of emotion regulation from general cognitive effort. However, this design choice aligns with standard practice in the field, where reappraisal is commonly compared to passive viewing conditions, and has been widely validated in both behavioral and pupillometry studies. A third limitation of the current study is the absence of a control group composed of individuals low in spider fear. Including such a group could have further clarified whether the observed changes in size estimation following emotion regulation were specific to individuals with high levels of fear, or reflect a more generalizable cognitive mechanism. However, this limitation is partly mitigated by findings from our previous and ongoing research, which include comparisons between individuals high and low in spider fear, as well as expert groups. These studies consistently demonstrate differential patterns of size estimation across groups, reinforcing the notion that perceptual biases are modulated by both emotional and experiential factors (Ben‐Baruch et al. 2022, 2025; Leibovich et al. 2016).

A fourth limitation concerns the fact that pupil dilation is a multifaceted physiological marker that can reflect both emotional arousal and cognitive effort. In the present study, we interpret the increased pupil diameter during reappraisal relative to passive viewing as indicative of regulation engagement, namely the recruitment of cognitive control processes required for emotion regulation. This interpretation is consistent with prior research employing similar paradigms (e.g., Kinner et al. 2017; Strauss et al. 2016). Future research may benefit from the inclusion of additional physiological measures (e.g., skin conductance or heart rate variability), which could help to further clarify the distinct roles of arousal and cognitive control in emotion regulation. Fifth, the study design was not optimal for assessing links between pupil dilation and behavioral findings, as was done in the mediation analysis. The task design of the two animals (spider and butterflies) in three cue conditions resulted in six trial types with 16 trials in each trial type. This did not allow the examination of the interaction between condition and animal on pupil size during the animal picture presentation. This number of trials may be enough for assessing the 2 × 3 interaction in the behavioral data, but not in the pupil data, in which some trials are removed due to missing data. This can be addressed in future studies.

To conclude, the findings of this study suggest that emotion regulation can effectively reduce perceptual biases related to fear‐inducing stimuli, such as spiders, as well as to neutral stimuli, such as butterflies. In the regulation condition, participants rated both spiders and butterflies as smaller, as compared to when exposed to negative images without regulation. Importantly, the consistent finding that spiders were rated as larger than butterflies, combined with the observed reduction in perceived spider size following the reappraise condition, suggests that size bias is influenced more by emotional processing and regulation than by the specific visual features of the stimuli. Previous research has shown that individuals with a high fear of spiders tend to overestimate spider size, a bias that was recently replicated in our laboratory (Ben‐Baruch et al. 2025). In contrast, spider experts provided the most accurate size estimations, supporting the notion that participants' judgments are guided more by conceptual knowledge than by purely visual characteristics. Our physiological data, showing increased pupil size during emotion regulation, support the notion that this strategy requires regulatory engagement. These results highlight the potential of emotion regulation techniques in mitigating perceptual distortions associated with negative emotions. Future research should investigate whether the effect of emotion regulation training on perceptual biases endures over time. This could involve assessing size perceptions of spiders following a series of daily reappraisal training sessions. Additionally, it would be valuable to explore whether other emotion regulation strategies, such as acceptance, can similarly reduce perceptual biases, akin to the effects observed with reappraisal.

## Author Contributions

**Noga Cohen:** conceptualization, validation, writing – review and editing, supervision. **Yahel Dror Ben‐Baruch:** conceptualization, investigation, writing – original draft, methodology, validation, visualization, writing – review and editing, software, formal analysis, project administration, data curation.

## Ethics Statement

The current study was approved by the Ethics Committee of Haifa University, approval number 059/19. At the beginning of the experiment, the participants signed a consent form to participate in the experiment according to the above wording: My name is Yahel Ben Baruch, and I am a PhD student at the Faculty of Education, in the Department of Special Education. I am reaching out to request your approval to participate in a study being conducted at the Special Education Lab at the Faculty of Education, University of Haifa. [This study is part of my PhD dissertation under the supervision of Dr. Noga Cohen]. The aim of the study is to examine the effect of emotion regulation on perception and pupil dilation. Participation in the study may benefit you by teaching you how to change negative emotions, and you will also be exposed to the process of conducting empirical research. As compensation for your participation, you will receive either credit points or payment. The study will take place in a lab and will last about an hour and a half. During the experiment, you will view negative images, which may cause temporary discomfort. You will also be asked to perform an emotion regulation task and estimate the size of animals (spiders and a butterflies). Throughout the experiment, we will use the Eyelink device to measure your pupil size. You will be asked to place your chin on a padded device, and we will ensure the device reads your pupils accurately and that you are seated comfortably. There will be no physical contact with your eyes, and pupil measurement will occur from a distance of several centimeters. Rest assured, no harm or risk will result from using the pupil measurement device. At the end of the experiment, you will be asked to complete questionnaires. It is important to note that participation in the study is only upon your signing of the consent form. This consent is voluntary, and you may withdraw from the study at any time without any negative consequences. Your personal details will be stored under a unique identifier only. Identifying details will be kept separately and will be permanently deleted once the necessary data processing for the study is completed. The data will not be shared in its raw form with any external parties, and access to the data will be limited to the research team. Research findings will be published in a way that ensures your anonymity. We would greatly appreciate your participation in the study. To confirm your participation, please sign the attached consent form and return it as soon as possible. If you would like more information about the study, feel free to contact us by phone or email. As a token of appreciation for your participation, you will receive 60 NIS for an hour of the experiment or 3 credit points. If you feel the need for it, you may retire from the research at any stage, without any harm being done to you. However, if you are unable to complete the experiment, the monetary compensation will be proportional to the time you participated, and not compensation in the full amount. I hereby declare that I agree to participate in the present study. I received an explanation of the study and its goals. I am aware of the fact that I am free to choose not to participate in the study and to terminate my participation in the experiment at any time, without my rights being violated, without any harm being done to me, and without any sanction being taken against me. I am guaranteed confidentiality regarding my personal identity at every stage of the research. I am guaranteed a willingness to answer any questions raised by me.

## Consent

The authors have nothing to report.

## Conflicts of Interest

The authors declare no conflicts of interest.

## Data Availability

The data that support the findings of this study are openly available in OSF at https://osf.io/et8au/.
